# Comparison of bone microstructures via high-resolution peripheral quantitative computed tomography in patients with different stages of chronic kidney disease before and after starting hemodialysis

**DOI:** 10.1080/0886022X.2022.2043375

**Published:** 2022-02-28

**Authors:** Kiyokazu Tsuji, Mineaki Kitamura, Ko Chiba, Kumiko Muta, Kazuaki Yokota, Narihiro Okazaki, Makoto Osaki, Hiroshi Mukae, Tomoya Nishino

**Affiliations:** a Department of Nephrology, Nagasaki University Graduate School of Biomedical Sciences, Nagasaki, Japan; b Department of Orthopedic Surgery, Nagasaki University Graduate School of Biomedical Sciences, Nagasaki, Japan; c Department of Respiratory Medicine, Nagasaki University Graduate School of Biomedical Sciences, Nagasaki, Japan

**Keywords:** Chronic kidney disease, renal insufficiency, bone microstructures, high-resolution peripheral quantitative computed tomography

## Abstract

Chronic kidney disease (CKD) negatively affects bone strength; however, the osteoporotic conditions in patients with CKD are not fully understood. Moreover, the changes in bone microstructure between pre-dialysis and dialysis are unknown. High-resolution peripheral quantitative computed tomography (HR-pQCT) reveals the three-dimensional microstructures of the bone. We aimed to evaluate bone microstructures in patients with different stages of CKD. This study included 119 healthy men and 40 men admitted to Nagasaki University Hospital for inpatient education or the initiation of hemodialysis. The distal radius and tibia were scanned with HR-pQCT. Patient clinical characteristics and bone microstructures were evaluated within 3 months of initiation of hemodialysis (in patients with CKD stage 5 D), patients with CKD stage 4–5, and healthy volunteers. Cortical bone parameters were lower in the CKD group than in healthy controls. Tibial cortical and trabecular bone parameters (cortical thickness, cortical area, trabecular volumetric bone mineral density, trabecular-bone volume fraction, and trabecular thickness) differed between patients with CKD stage 5 D and those with CKD stage 4–5 (*p* < 0.01). These differences were also observed between patients with CKD stage 5 and those with CKD stage 5 D (*p* < 0.017), but not between patients with CKD stage 4 and those with CKD stage 5, suggesting that the bone microstructure rapidly changed at the start of hemodialysis. Patients with CKD stage 5 D exhibited tibial microstructural impairment compared with those with CKD stage 4–5. HR-pQCT is useful for elucidation of the pathology of bone microstructures in patients with renal failure.

## Introduction

Chronic kidney disease (CKD) is associated with increased fracture risk. For instance, patients with CKD have an increased risk of hip fractures, and the hazard ratios for hip fracture were 1.89 (95% confidence interval [CI]: 1.47–2.43) and 3.75 (95% CI: 2.30–6.11) among patients with estimated glomerular filtration rates (eGFR) of 30–44 and 15–29 mL/min/1.73 m^2^, respectively, relative to those with an eGFR ≥ 60 mL/min/1.73 m^2^ [1]. Osteoporotic fractures, especially hip fractures, worsen patients’ limb movements, ability to perform activities of daily living, and quality of life, and even result in death [2–4].

Bone strength in patients with CKD is strongly associated with bone mineral density (BMD) and other factors such as bone quality. Structural properties include bone microstructure and geometry. Material properties include collagen, degree of calcification, and microdamage [5]. In patients with CKD, numerous additional factors affect bone strength, metabolic disorders (including secondary hyperparathyroidism and uremia) [6]. These factors interact with each other and complicate the bone conditions in patients with CKD [6]. However, these osteoporotic conditions in patients with CKD are unclear. Therefore, clarifying the detailed mechanisms of these conditions and their treatment are some of the most critical issues in daily practice in the field.

High-resolution peripheral quantitative computed tomography (HR-pQCT) is used for the in-vivo analysis of human limb bones and enables noninvasive, three-dimensional analysis of bone microstructures [7,8]. This technique enables analysis of the geometric, densitometric, microstructural, and mechanical properties of the trabecular and cortical bone structures of the distal radius and tibia [9,10]. Second-generation HR-pQCT (voxel size decreased from 82 to 61 μm) was introduced in 2014, allowing for a highly accurate evaluation of the bone microstructure. Early cross-sectional studies of the utility of dual-energy X-ray absorptiometry (DXA) for the discrimination of fractures in patients with end-stage renal disease did not discover significant associations between BMD and fractures [11–13]. Although several studies have revealed that DXA can be used to predict the risk of fracture in patients with CKD [14,15], such a two-dimensional assessment of BMD is insufficient to distinguish between cortical and trabecular bone. Compared to DXA, HR-pQCT can be used to generate axial images of the radius and tibia in which one can distinguish between cortical and trabecular bones. Moreover, HR-pQCT may be used to provide information on the risk of fractures, independent of DXA [16–20]. Indeed, previous studies with HR-pQCT have indicated that patients with CKD have thinner cortical bone and a lower trabecular bone mass than healthy controls [21–24]. However, we know of a few studies in which HR-pQCT was used for bone analysis across CKD stages [25,26].

Patients with CKD, especially those on dialysis, have an increased risk of fracture, and it is thought that their bone microstructures are affected by several factors, such as metabolic disorders. We hypothesized that a higher CKD stage would be associated with a higher degree of impairment in the bone microstructure. In this study, we aimed to evaluate bone microstructures by using HR-pQCT in patients with CKD according to their renal function.

## Materials and methods

### Patients

In this study, patients admitted to Nagasaki University Hospital for inpatient education or the initiation of maintenance hemodialysis between April 2017 and July 2019 were evaluated. Among them, patients for whom BMD and HR-pQCT data were available were included. As the bone quality in women is affected by menopause, we excluded all female patients.

In this study, we focused on the differences in bone microstructure among patients with different stages of CKD before and after starting hemodialysis. Additionally, we compared them with a healthy control group that consisted of age-matched volunteers without CKD to investigate the normal range of bone microstructural parameters. Patients were divided into two or three groups based on CKD stage and initiation of hemodialysis (stage 4–5 and stage 5 D; or stage 4, stage 5, and stage 5 D). CKD stage 4 was defined as having an eGFR of 15–29 mL/min/1.73 m^2^, and CKD stage 5 was defined as having an eGFR <15 mL/min/1.73 m^2^ (Kidney Disease: Improving Global Outcomes 2012 CKD guideline) [27]. Additionally, according to the median concentration of intact parathormone (PTH; high vs. low), patients were divided into two groups to evaluate the association between secondary hyperparathyroidism and bone microstructure parameters.

The educational program comprised the assessment of risk factors for CKD progression, clinical medication review, dietary and exercise guidance, and the introduction to renal replacement therapy. Patients with CKD stage 5 D received the same drug and dietary guidance, except for precautions during dialysis. The educational program was provided by a team of healthcare professionals comprising physicians, nurses, pharmacists, dietitians, and physical therapists. The decision of initiating hemodialysis was made by doctors who engaged in patients’ management, according to the general standards in Japan [28]. Patient geographic data, clinical history, etiology of renal failure, comorbidities, and treatment history were obtained from the medical records. This study was approved by the ethics committee of the Nagasaki University Hospital (Nagasaki, Japan; approval number 16042543), and written informed consent was obtained from all patients.

### Biochemical measurements

In addition to the general blood examinations for patients with renal failure, we evaluated bone turnover markers, such as tartrate-resistant acid phosphatase-5b (TRACP-5b) and procollagen type I N-terminal propeptide (PINP). Blood sampling was performed in the morning on the day of admittance for patients with CKD stage 4–5 (around 10 am) and when dialysis started for patients with CKD stage 5 D (around 9 am). Intact PTH, TRACP-5b, and PINP concentrations were measured with electrochemiluminescence immunoassay (ECLIA), enzyme immunoassay, and ECLIA, respectively. The TRACP-5b concentration was measured using the Osteolinks® TRACP-5b assay (Nittobo Medical, Fukushima, Japan). PINP was measured using the ECLUSIS SIYAKU total PINP^®^ assay (Roche Diagnostics K.K., Tokyo, Japan). All blood examinations were performed at the same time as HR-pQCT and DXA.

### Dual-energy X-ray absorptiometry

Areal BMD (aBMD) and the T-score in the lumbar spine (L1 to L4) and the proximal femur (total hip and femoral neck) were evaluated by using DXA (Lunar iDXA, GE Healthcare, Milwaukee, WI, USA).

### HR-pQCT

Bone microstructures of the distal radius and tibia of the non-dominant arm and leg were evaluated using HR-pQCT (Xtreme CT II, SCANCO Medical, Brüttisellen, Switzerland). If a patient had an arteriovenous fistula on the non-dominant arm, the evaluation was performed on the dominant arm. We used the data within three months after initiation of hemodialysis in the CKD 5 D group. The radial scan site was an area of the distal radius, 10.2 mm in width, 4% of the forearm length proximal from the hand joint. Furthermore, the tibial scan site was an area of the distal tibia, 10.2 mm in width, and 7.3% of the lower leg length proximal from the talocrural joint. The scanning conditions were as follows [29–31]: voltage, 68 kVp; tube current, 1470 μA; integration time, 4.3 ms; projection number, 900; field of view, 140 mm; matrix, 2304 × 2304; voxel size, 60.7 μm; scan length, 10.2 mm; and scanning time, 120 s. The computed tomography dose index, dose length product, and effective dose were 10.8 mGy, 11.0 mGy·cm, and 5 μSv, respectively. All images were evaluated for motion artifacts, and those with artifacts grade 3 or higher were excluded [32]. The semiautomatic algorithm was used for segmentation. For the periosteum, automatic contouring was performed with almost no manual correction. For the endosteum, however, automatic contouring was often followed by manual correction.

The following microstructure parameters were measured: cortical volumetric BMD (Ct.vBMD), cortical thickness (Ct.Th), cortical area (Ct.Ar), cortical perimeter, cortical porosity (Ct.Po), cortical pore diameter, trabecular volumetric BMD (Tb.vBMD), trabecular bone volume fraction (Tb.BV/TV), trabecular number (Tb.N), trabecular thickness (Tb.Th), and trabecular separation (Tb.Sp).

This study was conducted at a single center and evaluated with a single HR-pQCT. Phantoms were scanned every day for quality control.

### Statistical analyses

Categorical data are presented as numbers and percentages, and data of continuous parameters as means and standard deviations or medians and interquartile ranges. Continuous values were analyzed using the Wilcoxon rank-sum test, and categorical values were evaluated using Fisher’s exact test. The Kruskal–Wallis test was used for comparisons among three groups (CKD stage 4, 5, and 5 D), and post-hoc Bonferroni correction was applied. Patient clinical characteristics and bone microstructures were analyzed among three groups (CKD stages 4, 5, and 5 D). In addition, bone microstructural parameters were compared between two groups (CKD stages 4–5 and 5 D) to investigate whether renal failure affected the bone microstructure. We considered *p* < 0.05 statistically significant; however, a *p* < 0.017 was considered statistically significant in the post-hoc analysis. All statistical analyses were performed by using JMP Pro 14 (SAS Institute Inc., Cary, NC, USA).

## Results

Among the 40 patients with CKD enrolled in this study, 11 and 14 had CKD stages 4 and 5, respectively, and 15 had CKD stage 5 D (hemodialysis had been initiated). Table 1 summarizes the clinical characteristics of the 40 patients in these three groups, and we described the main characteristics for the two groups based on hemodialysis status (CKD stage 4–5 and 5 D). All patients were Japanese men. The mean ages in the CKD stage 4–5 (*n* = 25) and 5 D (*n* = 15) groups were 66.5 ± 9.9 and 61.1 ± 13.9 years (*p* = 0.16), respectively. No patient had undergone kidney transplantation. The prevalence of diabetes was 56% and 33% in the CKD stage 4–5 and 5 D groups (*p* = 0.16), respectively. According to interviews, none of the patients had a history of bone fractures.

**Table 1.** Characteristics of study participants.

Characteristic [normal range for men]	CKD stage 4 group (*n* = 11)	CKD stage 5 group (*n* = 14)	CKD stage 5D group (*n* = 15)	*p* Value
Age (years)	65.5 ± 8.5	67.2 ± 11.2	61.1 ± 13.9	0.16
Women	0 [0]	0 [0]	0 [0]	
Body weight (kg)	64.3 ± 7.0	67.3 ± 14.0	58.0 ± 12.4	0.06
BMI (kg/m^2^)	23.5 ± 3.1	24.3 ± 4.9	21.3 ± 3.4^a^	0.03
Hemoglobin (g/dL) [13.7–16.8]	12.6 ± 2.0	10.8 ± 1.3	9.9 ± 1.5^a^	0.008
Blood urea nitrogen (mg/dL) [8–20]	40.7 ± 13.8	65.5 ± 35.1	73 ± 30.8^a^	0.03
Serum creatinine (mg/dL) [0.65–1.07]	2.7 ± 0.4	5.1 ± 1.2	8.8 ± 3.9^c^	<0.001
eGFR (mL/min/1.73 m^2^)	20.0 ± 3.8	10.4 ± 2.8	6.5 ± 2.3^c^	<0.001
Serum albumin (g/dL) [4.1–5.1]	3.9 ± 0.3	3.4 ± 0.7	3.2 ± 0.7^a^	0.03
Serum calcium (mg/dL) [8.8–10.1]	8.7 ± 0.6	8.7 ± 0.8	8.2 ± 0.8	0.09
Serum phosphorus (mg/dL) [2.7–4.6]	3.5 ± 1.2	4.7 ± 1.1	5.6 ± 1.1^a^	0.001
Serum alkaline phosphatase (U/L) [106–322]	288 ± 92	250 ± 85	263 ± 117	0.71
Intact PTH (pg/mL) [15–65]	150 ± 63	254 ± 159	201 ± 143	0.83
TRACP-5b (mU/dL) [170–590]	555 ± 244	658 ± 301	585 ± 309	0.77
PINP (μg/L) [18.1–74.1]	78 (66–103)	91 (63–122)	168 (110–247)^a^	0.009
DXA total hip aBMD (g/cm^2^)	0.94 ± 0.1	0.98 ± 0.17	0.78 ± 0.12^c^	<0.001
DXA lumbar spine aBMD (g/cm^2^)	1.13 ± 0.18	1.23 ± 0.3	0.98 ± 0.13^b^	0.01
T–score total hip	–0.73 ± 0.75	–0.24 ± 1.25	–1.87 ± 0.8	<0.001
T–score lumbar spine	–0.09 ± 1.22	0.54 ± 2.2	–1.1 ± 1.0	0.048
Diabetes mellitus	7 [64]	7 [50]	5 [33]	0.16
Hypertension	10 [91]	13 [93]	12 [80]	0.28
Active vitamin D3 use	0 [0]	6 [43]	12 [80]	<0.001
Calcimimetic use	0 [0]	0 [0]	0 [0]	N/A
Parathyroidectomy	0 [0]	0 [0]	0 [0]	N/A
Bisphosphonate use	0 [0]	0 [0]	0 [0]	N/A
Current corticosteroid use	1 [9]	0 [0]	1 [7]	0.71
Calcium carbonate use	0 [0]	1 [7]	1 [7]	0.71
Other phosphate binder use	0 [0]	0 [0]	3 [20]	N/A

The Kruskal–Wallis test by rank and post-hoc Bonferroni correction were used. Data presented as *n* (%), mean ± standard deviation, or median (interquartile range).

^a^CKD stage 4 vs. CKD stage 5 D (*p* < 0.017).

^b^CKD stage 5 vs. CKD stage 5 D (*p* < 0.017).

^c^Both CKD stage 4 vs. CKD stage 5 D, and CKD stage 5 vs. CKD stage 5 D (*p* < 0.017).

aBMD: areal bone mineral density; BMI: body mass index; CKD: chronic kidney disease; DXA: dual-energy X-ray absorptiometry; eGFR: estimated glomerular filtration rate; PTH: parathormone; TRACP-5b: tartrate-resistant acid phosphatase-5b; PINP: procollagen type I N-terminal propeptide.

There were several statistically significant differences in the patient background characteristics among the three groups (CKD stage 4, 5, and 5 D) and between the two groups (CKD stage 4–5 and 5 D). For example, activated vitamin D analogs were prescribed more commonly in the CKD stage 5 D group (80%) than in the CKD stage 4–5 group (24%). Additionally, hemoglobin concentration, serum albumin concentration, and body mass index were lower, while serum creatinine and phosphorus concentrations were higher in the CKD stage 5 D group than in the CKD stage 4 group (both *p* < 0.017). However, of those differences, only serum creatinine differed between the CKD stage 5 D and CKD stage 5 group (*p* < 0.017). There were no statistically significant differences in phosphate binders or bisphosphonate use among the groups. Furthermore, serum calcium, intact PTH, and TRACP-5b concentrations did not differ among the groups. Two patients were prescribed corticosteroids: one because of cholesterol embolism (CKD stage 4) and one because of anti-glomerular basement membrane disease (CKD stage 5 D). DXA aBMDs were lower in the CKD stage 5 D group than in the CKD stage 4 group (total hip, *p* < 0.01) and in the CKD stage 5 group (total hip, *p* < 0.01). DXA lumbar spinal aBMD did not differ between the CKD stage 4 group and the CKD stage 5 D group (*p* = 0.05).

HR-pQCT parameters were first compared between the CKD (*n* = 40) and the healthy control groups (aged 64.8 ± 9.9 years) (*n* = 119) to investigate the normal range of bone microstructural parameters (Tables 2 and 3). The comparison of the characteristics of healthy controls and patients with CKD is summarized in Supplementary Table S1. Generally, HR-pQCT parameters of the cortical bone in the tibia and radius were statistically significantly lower in the CKD group than in the healthy control group, but those of the trabecular bone in the tibia did not differ between these groups.

**Table 2.** Comparison of HR-pQCT parameters in the distal tibia between healthy control and CKD groups.

Parameter	Healthy control group (*n* = 119)	CKD group (*n* = 40)	*p* Value
Total vBMD (mg/cm^3^)	291 ± 47	264 ± 59	0.005
Ct.vBMD (mg/cm^3^)	864 ± 55	833 ± 79	0.04
Ct.Th (mm)	1.55 ± 0.26	1.34 ± 0.31	<0.001
Ct.Ar (mm^2^)	146 ± 24	123 ± 30	<0.001
Ct.Pm (mm)	112 ± 7	108 ± 11	0.06
Ct.Po (%)	3.8 ± 1.6	3.8 ± 2.0	0.9
Ct.Po.Dm (mm)	0.24 ± 0.03	0.23 ± 0.03	0.005
Tb.vBMD (mg/cm^3^)	166 ± 32	154 ± 44	0.13
Tb.BV/TV (%)	25 ± 4	23 ± 6	0.11
Tb.N (1/mm)	1.22 ± 0.15	1.21 ± 0.19	0.64
Tb.Th (mm)	0.26 ± 0.02	0.25 ± 0.02	0.31
Tb.Sp (mm)	0.79 ± 0.10	0.82 ± 0.18	0.49

The Wilcoxon rank-sum test was used.

Data presented as mean ± standard deviation.

CKD: chronic kidney disease; vBMD: volumetric bone mineral density; Ct.vBMD: cortical volumetric bone mineral density; Ct.Th: cortical thickness; Ct.Ar: cortical area; Ct.Pm: cortical perimeter; Ct.Po: cortical porosity; Ct.Po.Dm: cortical pore diameter; Tb.vBMD: trabecular volumetric bone mineral density; Tb.BV/TV: trabecular bone volume fraction; Tb.N: trabecular number; Tb.Th: trabecular thickness; Tb.Sp: trabecular separation.

**Table 3.** Comparison of HR-pQCT parameters in the distal radius between healthy control and CKD groups.

Parameter	Healthy control group (*n* = 119)	CKD group (*n* = 40)	*p* Value
Total vBMD (mg/cm^3^)	291 ± 48	281 ± 63	0.59
Ct.vBMD (mg/cm^3^)	863 ± 55	865 ± 71	0.55
Ct.Th (mm)	1.52 ± 0.29	1.06 ± 0.22	<0.001
Ct.Ar (mm^2^)	142 ± 30	71 ± 19	<0.001
Ct.Pm (mm)	110 ± 10	82 ± 11	<0.001
Ct.Po (%)	3.8 ± 1.8	1.4 ± 0.8	<0.001
Ct.Po.Dm (mm)	0.24 ± 0.03	0.20 ± 0.03	<0.001
Tb.vBMD (mg/cm^3^)	166 ± 32	145 ± 44	0.02
Tb.BV/TV (%)	25 ± 4	21 ± 6	0.002
Tb.N (1/mm)	1.23 ± 0.16	1.25 ± 0.22	0.46
Tb.Th (mm)	0.25 ± 0.02	0.23 ± 0.02	<0.001
Tb.Sp (mm)	0.79 ± 0.10	0.79 ± 0.18	0.37

The Wilcoxon rank-sum test was used.

Data presented as mean ± standard deviation.

CKD: chronic kidney disease; vBMD: volumetric bone mineral density; Ct.vBMD: cortical volumetric bone mineral density; Ct.Th: cortical thickness; Ct.Ar: cortical area; Ct.Pm: cortical perimeter; Ct.Po: cortical porosity; Ct.Po.Dm: cortical pore diameter; Tb.vBMD: trabecular volumetric bone mineral density; Tb.BV/TV: trabecular bone volume fraction; Tb.N: trabecular number; Tb.Th: trabecular thickness; Tb.Sp: trabecular separation.

In order to evaluate the association between secondary hyperparathyroidism and bone microstructural parameters, patients with CKD were divided into two groups according to the median concentration of intact PTH (179 pg/mL): high (*n* = 19, average = 311.5 pg/mL) and low (*n* = 20, average = 107.5 pg/mL). Intact PTH level was not measured in one patient. Most bone microstructural parameters did not differ between these groups and only Tb.Sp was lower in patients with high concentrations of intact PTH (*p* = 0.01).

The comparisons of the HR-pQCT parameters between the CKD stage 4–5 5 D groups are summarized in Tables 4 and 5. In the CKD stage 5 D group, the mean Ct.Th, Tb.vBMD, Tb.BV/TV, and Tb.Th in the tibia were 1.18 mm, 122 mg/cm^3^, 18%, and 0.24 mm, respectively. Each of these values was lower than the corresponding values in the CKD stage 4–5 group (*p* < 0.01). There were no statistically significant differences in Ct.vBMD, Ct.Po, and Tb.N between the two groups. Additionally, there was statistically significant difference in the Ct Ar of the radius between these two groups (Table 5).

**Table 4.** Comparison of HR-pQCT parameters in the distal tibia between CKD stage 4–5 and CKD stage 5 D groups.

Parameter	CKD stage 4–5 group (*n* = 25)	CKD stage 5D group (*n* = 15)	*p* Value
Total vBMD (mg/cm^3^)	290 ± 51	221 ± 43	<0.001
Ct.vBMD (mg/cm^3^)	842 ± 73	818 ± 89	0.39
Ct.Th (mm)	1.45 ± 0.29	1.18 ± 0.28	0.009
Ct.Ar (mm^2^)	133 ± 27	108 ± 29	<0.001
Ct.Pm (mm)	109 ± 9	107 ± 13	0.96
Ct.Po (%)	3.8 ± 1.8	3.8 ± 2.2	0.71
Ct.Po.Dm (mm)	0.23 ± 0.03	0.23 ± 0.04	0.56
Tb.vBMD (mg/cm^3^)	173 ± 34	122 ± 39	<0.001
Tb.BV/TV (%)	26 ± 5	18 ± 5	<0.001
Tb.N (1/mm)	1.24 ± 0.18	1.15 ± 0.21	0.29
Tb.Th (mm)	0.26 ± 0.02	0.24 ± 0.02	0.001
Tb.Sp (mm)	0.79 ± 0.11	0.89 ± 0.24	0.16

The Wilcoxon rank-sum test was used.

Data presented as mean ± standard deviation.

CKD: chronic kidney disease; vBMD: volumetric bone mineral density; Ct.vBMD: cortical volumetric bone mineral density; Ct.Th: cortical thickness; Ct.Ar: cortical area; Ct.Pm: cortical perimeter; Ct.Po: cortical porosity; Ct.Po.Dm: cortical pore diameter; Tb.vBMD: trabecular volumetric bone mineral density; Tb.BV/TV: trabecular bone volume fraction; Tb.N: trabecular number; Tb.Th: trabecular thickness; Tb.Sp: trabecular separation.

**Table 5.** Comparison of HR-pQCT parameters in the distal radius between stage CKD 4–5 and CKD stage 5 D groups.

Parameter	CKD stage 4–5 group (*n* = 25)	CKD stage 5D group (*n* = 15)	*p* Value
Total vBMD (mg/cm^3^)	287 ± 59	272 ± 70	0.37
Ct.vBMD (mg/cm^3^)	874 ± 47	851 ± 97	0.53
Ct.Th (mm)	1.11 ± 0.19	0.99 ± 0.25	0.11
Ct.Ar (mm^2^)	77 ± 21	64 ± 12	0.02
Ct.Pm (mm)	83 ± 9	82 ± 14	0.42
Ct.Po (%)	1.5 ± 0.7	1.3 ± 0.8	0.40
Ct.Po.Dm (mm)	0.20 ± 0.03	0.20 ± 0.02	1.00
Tb.vBMD (mg/cm^3^)	154 ± 43	132 ± 44	0.19
Tb.BV/TV (%)	22 ± 6	19 ± 6	0.16
Tb.N (1/mm)	1.29 ± 0.22	1.18 ± 0.21	0.13
Tb.Th (mm)	0.24 ± 0.02	0.23 ± 0.01	0.20
Tb.Sp (mm)	0.75 ± 0.18	0.84 ± 0.19	0.13

The Wilcoxon rank-sum test was used.

Data presented as mean ± standard deviation.

CKD: chronic kidney disease; vBMD: volumetric bone mineral density; Ct.vBMD: cortical volumetric bone mineral density; Ct.Th: cortical thickness; Ct.Ar: cortical area; Ct.Pm: cortical perimeter; Ct.Po: cortical porosity; Ct.Po.Dm: cortical pore diameter; Tb.vBMD: trabecular volumetric bone mineral density; Tb.BV/TV: trabecular bone volume fraction; Tb.N: trabecular number; Tb.Th: trabecular thickness; Tb.Sp: trabecular separation.

**Figure 1.**
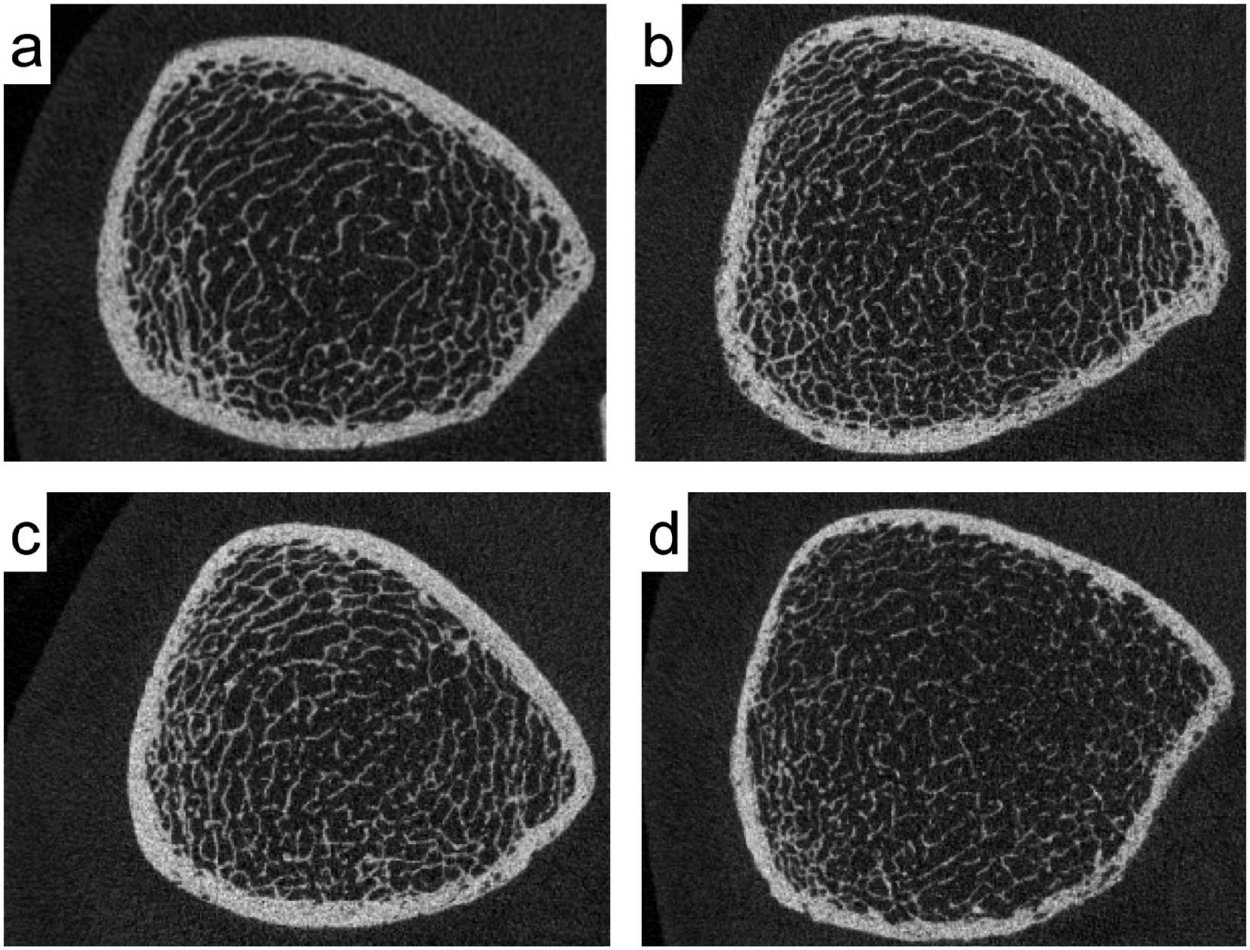
HR-pQCT images of the distal tibia were used to evaluate the mean cortical thickness (Ct.Th) and trabecular volumetric bone mineral density (Tb.vBMD) in healthy controls and in patients with CKD of each stage. (a) A 68-year-old man without CKD. (b) A 63-year-old man with CKD stage 4. (c) A 74-year-old man with CKD stage 5. (d) A 59-year-old man with CKD stage 5D. Compared to healthy controls, patients with CKD exhibited thinning of the cortical bone and osteoporotic changes. In addition to thinning of the cortical bone, osteoporotic changes in the trabecular bone are more pronounced in patients with CKD stage 5D (d) than in those with CKD stages 4 and 5 (b, c).

Furthermore, we compared the tibial parameters among the CKD stages 4, 5, and 5 D groups. The results are summarized in Table 6. The mean Ct.Th, Tb.vBMD, Tb.BV/TV, and Tb.Th in these groups were 1.47, 1.42, and 1.18 mm, respectively; 164, 180, and 122 mg/cm^3^, respectively; 25%, 27%, and 18%, respectively; and 0.26, 0.26, and 0.24 mm, respectively (*p* < 0.05). Figure 1 contains representative HR-pQCT images of the distal tibia for the mean Ct.Th and Tb.vBMD in each group. Tb.vBMD, Tb.BV/TV, and Tb.Th were lower in the CKD stage 5 D group than in the CKD stage 4 and 5 groups (*p* < 0.017).

**Table 6.** Comparison of HR-pQCT parameters in the distal tibia among the three groups.

	CKD stage 4 group (*n* = 11)	CKD stage 5 group (*n* = 14)	CKD stage 5D group (*n* = 15)	*p* Value
Total vBMD (mg/cm^3^)	281 ± 37	297 ± 61	222 ± 43^c^	<0.001
Ct.vBMD (mg/cm^3^)	834 ± 51	849 ± 88	818 ± 89	0.58
Ct.Th (mm)	1.47 ± 0.20	1.42 ± 0.35	1.18 ± 0.28^a^	0.02
Ct.Ar (mm^2^)	132 ± 22	133 ± 30	108 ± 29^b^	0.03
Ct.Pm (mm)	108 ± 11	109 ± 8	107 ± 13	0.94
Ct.Po (%)	4.0 ± 1.4	3.6 ± 2.1	3.8 ± 2.2	0.89
Ct.Po.Dm (mm)	0.23 ± 0.03	0.23 ± 0.03	0.23 ± 0.04	0.83
Tb.vBMD (mg/cm^3^)	164 ± 24	180 ± 40	122 ± 39^c^	<0.001
Tb.BV/TV (%)	25 ± 3	27 ± 5	18 ± 5^c^	<0.001
Tb.N (1/mm)	1.19 ± 0.10	1.28 ± 0.21	1.15 ± 0.21	0.16
Tb.Th (mm)	0.26 ± 0.02	0.26 ± 0.02	0.24 ± 0.02	0.02
Tb.Sp (mm)	0.82 ± 0.07	0.76 ± 0.14	0.89 ± 0.24	0.16

^a^CKD stage 4 vs. CKD stage 5 D (*p* < 0.017).

^b^CKD stage 5 vs. CKD stage 5 D (*p* < 0.017).

^c^Both CKD stage 4 vs. 5 D and 5 vs. 5 D (*p* < 0.017).

Data presented as mean ± standard deviation.

CKD: chronic kidney disease; vBMD: volumetric bone mineral density; Ct.vBMD: cortical volumetric bone mineral density; Ct.Th: cortical thickness; Ct.Ar: cortical area; Ct.Pm: cortical perimeter; Ct.Po: cortical porosity; Ct.Po.Dm: cortical pore diameter; Tb.vBMD: trabecular volumetric bone mineral density; Tb.BV/TV: trabecular bone volume fraction; Tb.N: trabecular number; Tb.Th: trabecular thickness; Tb.Sp: trabecular separation.

## Discussion

Patients with CKD, especially those on dialysis, have an increased risk of fracture, and it is thought that their bone microstructure is affected by several factors such as metabolic disorders. In this study, we evaluated the bone microstructure by using HR-pQCT in patients with renal failure according to their renal function. Consequently, patients with CKD stage 5 D had lower tibial values for Ct.Th, Tb.vBMD, Tb.BV/TV, and Tb.Th than those with CKD stage 4–5, indicating microstructural impairments in both the cortical and trabecular bones. These differences were observed only in the tibia, a weight-bearing bone, but not in the radius.

A recent study revealed that the risk of bone fractures increases with the severity of renal dysfunction and is pronounced even in the earlier stages of CKD (eGFR of 45–59 mL/min/1.73 m^2^) [33]. In one survey, the prevalence of hip fractures among patients with CKD was 5.2% [34]. Moreover, the risk of hip fractures was especially increased in patients on dialysis [35]. In an international cohort, the prevalence of bone fracture among patients on hemodialysis was higher than that in healthy individuals in every country [36]. A meta-analysis has demonstrated that the HR of hip fracture in patients on dialysis was 4.92 (95% CI: 4.30–5.63) and that of any type of fracture in patients with an eGFR <15 mL/min/1.73 m^2^ was 2.63 (95% CI: 1.74–3.98) compared with those in healthy controls [35].

Generally, in addition to BMD, bone strength in patients with CKD may be strongly affected by other factors, such as bone quality; the latter is defined by both structural and material strength. In contrast, a previous report revealed no statistically significant association between PTH concentration and hip fractures [37]. It is believed that overexpression of sclerostin in bone cells because of uremia plays an important role in the low turnover of bone despite a high PTH concentration [10,38,39]. Although there were no statistically significant differences in TRACP-5b and intact PTH concentrations among the groups, the PINP concentration increased with the progression of renal failure in this study. Consequently, bone changes in patients with renal failure may be associated with alterations in collagen deposition. However, renal insufficiency results in deterioration of the elastic mechanical properties of bones irrespective of bone metabolism or mass [40]. Therefore, bone lesions in patients with CKD are caused by multiple factors.

The most important predictive factor for bone fracture in a previous study was Tb.BV/TV of the tibia [22]. In another study, the strongest predictive factor for bone strength was Ct.Th [30]. Both these factors were impaired in the CKD patients of this study. Additionally, the structural features of cortical bones are reflected by Ct.Ar and Ct.Th. Generally, cortical bones in patients with CKD tend to become thin and porous, which may be caused by secondary hyperparathyroidism [41]. In this study, there were statistically significant differences in Ct.Ar and Ct.Th between the healthy control group and the CKD group. Cortical bone microstructural parameters, such as Ct.Ar, Ct.Th in the tibia, appeared lower in patients with CKD stage 5 D than in those with stages 4 or 5, but there was no statistically significant difference. The lack of decline in these parameters from CKD stage 4 to 5 D may be because cortical bone parameters are impaired from the early CKD stages by secondary hyperparathyroidism [42]. However, no statistically significant correlations were detected for any of the microstructural parameters and intact PTH concentration among our patients with CKD. A previous report demonstrated that slowly progressive renal disease was more frequently associated with osteomalacia or mixed renal osteodystrophy than with glomerulonephritis (which is rapidly progressive) [43] and that the duration of CKD rather than the intact PTH concentration might negatively affect the bone microstructure. However, the duration of CKD complicated with secondary hyperparathyroidism was unknown for several cases in this study, as certain patients were not aware that they had developed renal failure. Therefore, we could not analyze the duration of CKD combined with secondary hyperparathyroidism.

Remarkably, there were no statistically significant differences in Tb.vBMD, Tb.BV/TV, and Tb.Th in the tibia between the healthy control group and CKD group. This might have been because, even in healthy people, the trabecular bone in the tibia exhibits a more pronounced age-dependent decrease than that in the radius [44]. These trabecular microstructural parameters in the tibia were lower in patients with CKD stage 5 D than in those with CKD stages 4 and 5, and there were statistically significant differences in Tb.vBMD and Tb.BV/TV either. In the later stages, such as during the initiation of hemodialysis and during the maintenance period, the prevalence of mixed osteodystrophy, low-turnover bone disease, and osteomalacia are known to increase [42,45]. Additionally, it is believed that uremia impairs bone quality [46]. Therefore, the differences in the trabecular microstructural parameters between the CKD stage 4–5 and 5 D groups may be explained by uremia occurring just before the initiation of dialysis.

Cortical bone parameters in the radius are associated with bone fractures [13,21]. Compared with healthy people, patients with CKD demonstrated a statistically significantly lower aBMD, Ct.Th, and Tb.N in the radius in our study. However, the impaired microstructures in the tibia, a weight-bearing bone, were more noticeable than those in the radius among the CKD groups. Lower BMD in weight-bearing bones is thought to be associated with immobility. The prevalence of frailty is reported to increase with a decrease in the eGFR [47,48]. Moreover, patients with CKD on dialysis have a higher prevalence of frailty than those not on dialysis [49]. Furthermore, the functional decline of patients before and after initiation of hemodialysis is common [50,51]. Immobility due to frailty before and after the initiation of hemodialysis may reduce the load on weight-bearing bones and play an important role in the deterioration of the tibial microstructure in patients with CKD stage 5 D.

This study has several limitations. First, the sample size was relatively small. Unfortunately, the in-hospital education program was suspended at the start of the COVID-19 pandemic, and we could not increase the number of patients. This was a proof-of-concept study, and its results should be validated in a larger cohort as HR-pQCT becomes more widely used. Second, this was a cross-sectional study, which is a critical drawback. Hence, we could not observe the time-dependent deterioration of bone quality in the same patients. Moreover, we could not evaluate the association between the duration of the abnormal altered laboratory parameters and the bone microstructure. Therefore, longitudinal studies are needed in the future. Third, although HR-pQCT can be used to assess bone microarchitecture, it cannot be used to distinguish between the many forms of CKD-mineral and bone disorders, including osteoporosis, osteomalacia, secondary hyperparathyroidism, and adynamic bone disease. Furthermore, the risk factors for osteoporosis in men, including physical activities, testosterone concentration, smoking, excess consumption of alcohol, serum vitamin D levels, and total intake of calcium, were not evaluated [52,53]. Generally, pelvis/hip, vertebral, and lower leg fractures are the most prevalent fracture types in patients undergoing hemodialysis [54]. However, the most common fracture sites other than the tibia could not be assessed with HR-pQCT. Women were excluded as menopause strongly affects BMD. Consequently, the results in this study cannot be applied to women with similar conditions. Finally, we did not perform radiologic exams to evaluate past bone fractures. We only interviewed the patients regarding past bone fractures, introducing the possibility of recall bias.

## Conclusion

Bone microstructural changes were evaluated using HR-pQCT in patients with different stages of CKD. Patients with CKD stage 5 D had statistically significant impairments in Ct.Th, Tb.vBMD, Tb.BV/TV, and Tb.Th in the tibia compared with those with CKD stages 4 and 5. The microstructures in both cortical and trabecular bone in the tibia were altered in patients with more advanced stages of CKD relative to those with earlier stages of CKD. The changes in bone quality in patients with renal failure may be determined by renal failure-specific factors, such as secondary hyperparathyroidism, uremia, and their duration. Longitudinal studies with HR-pQCT are needed to elucidate the pathology of bone microstructures in patients with renal failure.
